# Elevated CO_2_ and Nitrogen Supply Boost N Use Efficiency and Wheat (*T. aestivum* cv. Yunmai) Growth and Differentiate Soil Microbial Communities Related to Ammonia Oxidization

**DOI:** 10.3390/plants13172345

**Published:** 2024-08-23

**Authors:** Xingshui Dong, Hui Lin, Feng Wang, Songmei Shi, Sharifullah Sharifi, Shuai Wang, Junwei Ma, Xinhua He

**Affiliations:** 1 Centre of Excellence for Soil Biology, School of Resource and Environment, Southwest University, Chongqing 400715, China; xingshuid@outlook.com (X.D.); 2022020@ynau.edu.cn (S.S.); nsharifullah@gmail.com (S.S.); shuaiwang@swu.edu.cn (S.W.); 2 State Key Laboratory for Managing Biotic and Chemical Threats to the Quality and Safety of Agro-Products, Institute of Environment, Resource, Soil and Fertilizers, Zhejiang Academy of Agricultural Sciences, Hangzhou 310021, China; linhui@zaas.ac.cn (H.L.); wangfeng@zaas.ac.cn (F.W.); 3 Department of Land, Air and Water Resources, University of California at Davis, Davis, CA 90616, USA; 4 School of Biological Sciences, University of Western Australia, Perth 6009, Australia

**Keywords:** nitrogen accumulation, *Nitrosomonadaceae*, *Nitrosospira*, *Nitrosomonas*, *Triticum aestivum* L.

## Abstract

Elevated CO_2_ levels (eCO_2_) pose challenges to wheat (*Triticum aestivum* L.) growth, potentially leading to a decline in quality and productivity. This study addresses the effects of two ambient CO_2_ concentrations (aCO_2_, daytime/nighttime = 410/450 ± 30 ppm and eCO_2_, 550/600 ± 30 ppm) and two nitrogen (N) supplements (without N supply—N0 and with 100 mg N supply as urea per kg soil—N100) on wheat (*T. aestivum* cv. Yunmai) growth, N accumulation, and soil microbial communities related to ammonia oxidization. The data showed that the N supply effectively mitigated the negative impacts of eCO_2_ on wheat growth by reducing intercellular CO_2_ concentrations while enhancing photosynthesis parameters. Notably, the N supply significantly increased N concentrations in wheat tissues and biomass production, thereby boosting N accumulation in seeds, shoots, and roots. eCO_2_ increased the agronomic efficiency of applied N (AE_N_) and the physiological efficiency of applied N (PE_N_) under N supply. Plant tissue N concentrations and accumulations are positively related to plant biomass production and soil NO_3_^−^-N. Additionally, the N supply increased the richness and evenness of the soil microbial community, particularly *Nitrososphaeraceae*, *Nitrosospira*, and *Nitrosomonas*, which responded differently to N availability under both aCO_2_ and eCO_2_. These results underscore the importance and complexity of optimizing N supply and eCO_2_ for enhancing crop tissue N accumulation and yield production as well as activating nitrification-related microbial activities for soil inorganic N availability under future global environment change scenarios.

## 1. Introduction

As CO_2_ is a fundamental component of photosynthesis, its increase can initially seem beneficial, promoting faster growth and potentially higher yields in various plant species [1]. This suggests a promising aspect for promoting agricultural productivity, yet the benefits are shadowed by complex challenges (IPCC, 2021) [2]. Elevated CO_2_ (eCO_2_) levels (2.0 to 2.4 ppm per year since 2000) contribute to global warming, altering weather patterns and threatening food security through the potential loss of crop diversity and productivity in most regions [3]. Furthermore, the nutritional quality of crop produce can be diminished under higher CO_2_ conditions, impacting human health by reducing the availability of essential nutrients in staple foods [4,5]. Therefore, understanding the relationship among CO_2_, plant growth, and human living necessitates a nuanced approach, acknowledging both the potential short-term gains in agricultural productivity and the long-term implications or risks for human well-being and environmental sustainability [2,6,7].

Wheat (*Triticum aestivum* L.) stands as one of the three primary cereals of global agricultural economy and food security since it serves as a primary food source for a significant portion of the world’s population [8]. Cultivated in a diverse range of climates and regions, wheat varieties are incredibly adaptable, with growth regions ranging from the sunny fields of the Mediterranean to the colder, temperate zones of Asia, Australasia, Europe, and North America [8]. This adaptability has made wheat not only a staple food but also a versatile ingredient found in an array of products, such as bread, pasta, and cereals. However, wheat production faces challenges, including climate change, pests, diseases, and the need for sustainable farming practices to ensure that this vital crop can continue to feed the ever-increasing global population [8]. Elevated CO_2_ enhances photosynthesis and water efficiency in wheat but reduces stomatal conductance, nitrogen (N), and grain quality, with cultivar-dependent impacts on yield and nutrient content, offering insights for future cultivation strategies [5,9,10]. Under conditions of ample N supply, plants exposed to eCO_2_ can effectively absorb N, maintaining minimal losses of this nutrient through foliar processes [11]. However, higher N application rates do not mean higher N use efficiency [12].

High N levels can alter soil microbial communities and reduce biodiversity by favoring certain species over others, potentially disrupting natural soil processes and ecosystem services [13]. Nitrogen supplies, especially those high in ammonium or urea, can increase soil acidity, leading to a decrease in soil pH, which can affect the availability of other nutrients and the activity of soil microorganisms [14]. To address environmental changes, such as eCO_2_, soil microorganisms can adjust by modifying their metabolic activities and population structures [15]. These adaptations subsequently influence plant growth and the overall well-being of the soil ecosystem. Observations have indicated that eCO_2_ can result in a notable decrease in soil NO_3_^−^-N concentrations, potentially attributable to enhanced plant absorption or losses through leaching into groundwater and emissions into the atmosphere [16,17,18]. Elevated CO_2_ also influences the soil microbial community indirectly through alterations in plant metabolism and root exudates, with a notable impact observed in C_3_ plant species [19]. There are limited understandings of how eCO_2_ impacts the structure and, more importantly, the function of rhizosphere and root-surface-associated microbial communities. Current research primarily has focused on the structure of rhizosphere microbial communities, with less attention given to those associated directly with the root surface [19,20,21]. When N availability is abundant, microbial activity and N utilization are boosted by higher CO_2_ levels. This has substantial implications for comprehending the effects of eCO_2_ on C and N dynamics within ecosystems [22,23]. Previous research has demonstrated that when N is supplied, the activity and abundance of denitrifiers possessing *nirS*, *nirK*, and *nosZ* genes are notably influenced in the wheat rhizosphere [24]. Under eCO_2_, there is a marked increase in the population of ammonia-oxidizing archaea (AOA) and ammonia-oxidizing bacteria (AOB), which significantly enhances the nitrification potential in topsoil [25]. The interplay between eCO_2_ and human-induced N inputs has a notable impact on the dynamics of soil microbial communities and their functioning, which can lead to shifts in microbial diversity, activity, and the ecological roles that they perform, ultimately influencing soil fertility and plant growth [26,27,28]. Meanwhile, eCO_2_ may hinder the microbial process, potentially by disrupting the microbial capacity for intracellular electron transport and utilization [29].

As atmospheric CO_2_ concentrations continuously increase, it becomes crucial to reassess N fertilization practices to optimize soil and crop management. By doing so, we can enhance agricultural yields by leveraging the benefits of higher CO_2_ levels, ensure the maintenance of crop quality, and reduce the environmental risks associated with N runoff and leaching. Current indoor studies lack the ability to fully account for the comprehensive impacts of environmental factors or the specific conditions found in actual field settings. Consequently, we implemented an automated system to control environmental factors (e.g., similar light, temperature, and humidity were automatically adjusted inside and outside the glass growth chamber), allowing us to monitor both plant and soil characteristics [30,31]. This system measures changes in plant growth parameters, soil physicochemical properties, and soil microbial community composition. We hypothesized that (1) eCO_2_ and N supply could increase N concentrations in wheat tissues, and (2) the soil microbial community would be altered under eCO_2_ and/or N supply. The present study aimed to boost wheat yield and quality while providing a theoretical foundation for a rational N fertilization strategy in the context of rising CO_2_ concentrations and altered soil microbial communities. The results generated from this study would then advise agricultural practices, helping manage soil microorganisms for effective adaptation to climate change, thereby promoting agricultural sustainability.

## 2. Results

### 2.1. Variation in Plant Growth Characteristics

**Figure 1:**
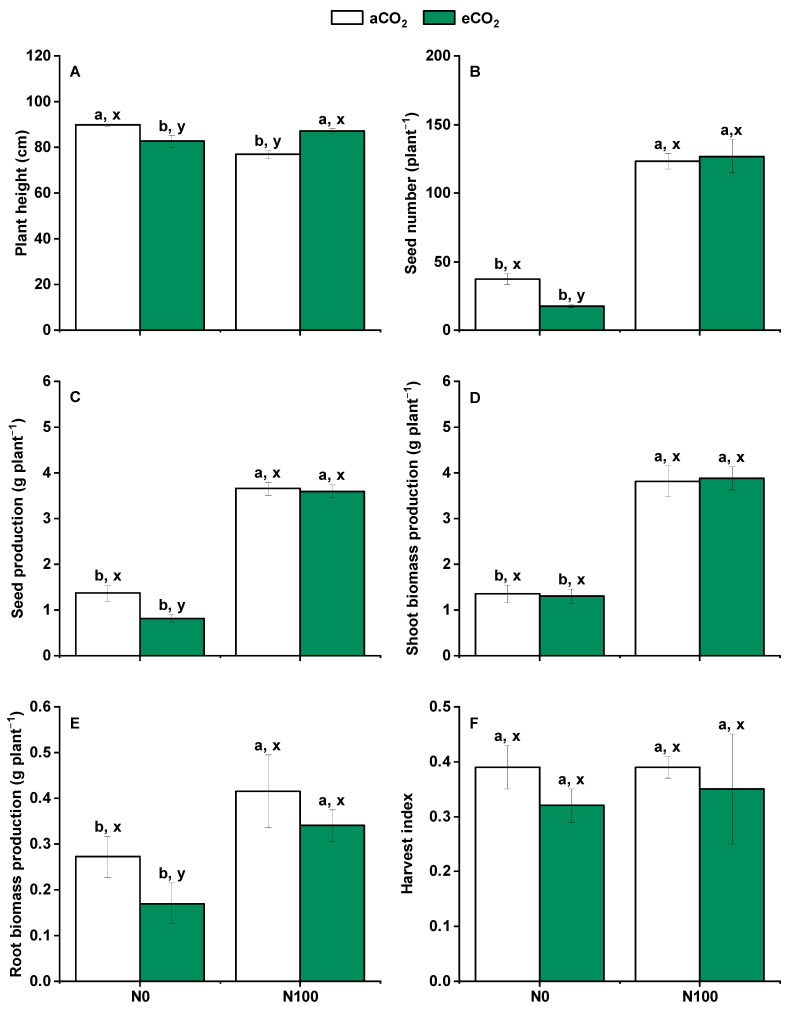
Effects of CO_2_ and N supply on 5-month-old wheat at harvest. (**A**) Plant height; (**B**) Seed number; (**C**) Seed production; (**D**) Shoot (stem and leaf) biomass production; (**E**) Root biomass production; (**F**) Harvest index = seed production/shoot biomass. Data are means ± SE (*n* = 3). Lower-case letters above the bars indicate a significant difference between N supplies for the same aCO_2_ treatment (a, b) or eCO_2_ treatment (**A**,**B**) and between CO_2_ concentrations for the same N treatment (x, y) at *p* < 0.05. Abbreviations: aCO_2_, atmospheric CO_2_; eCO_2_, elevated CO_2_; N0, no N supply; N100, 100 mg N kg^−1^ DW soil.

Under aCO_2_, N100 significantly increased seed number, seed production, and shoot biomass and root biomass production (Figure 1B–E) and significantly decreased the height of wheat (Figure 1A). Under eCO_2_, N100 significantly increased the height of wheat, seed number, seed production, and shoot biomass and root biomass production. Elevated CO_2_ and N input did change the harvest index (Figure 1). Elevated CO_2_ significantly decreased plant height, seed number, seed production, and root biomass production under N0, while N100 prevented these reductions (Figure 1A–C,E).

### 2.2. Variation in Basic Photosynthetic Characteristics

**Figure 2:**
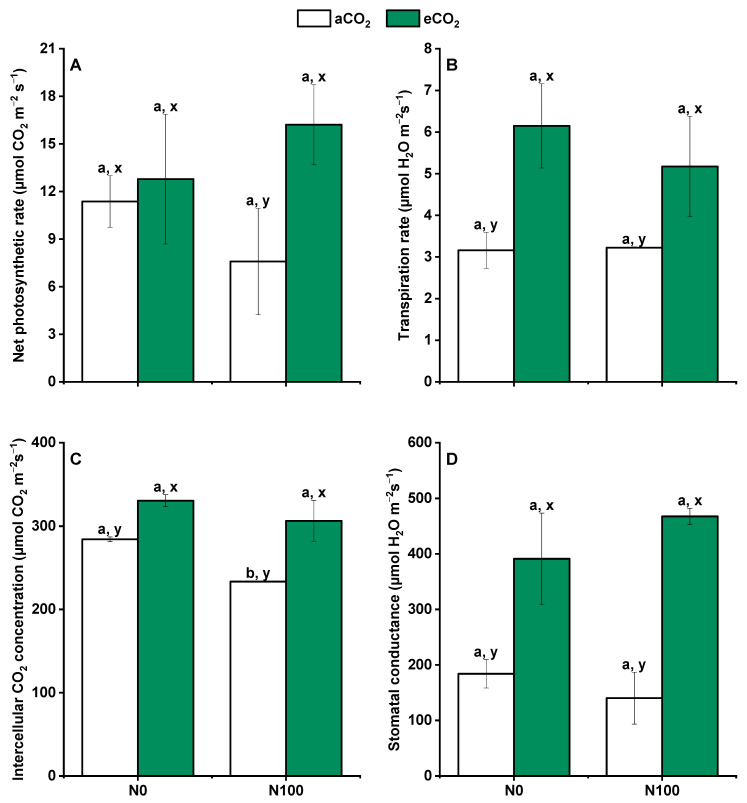
Effects of CO_2_ and N supply on photosynthesis parameters during the booting stage of 5-month-old wheat. (**A**) Net photosynthetic rate; (**B**) Transpiration rate; (**C**) Intercellular CO_2_ concentration; (**D**) Stomatal conductance. Data are means ± SE (*n* = 3). Lower-case letters above the bars indicate a significant difference between N supplies for the same aCO_2_ treatment (a, b) or eCO_2_ treatment (**A**,**B**) and between CO_2_ concentrations for the same N treatment (x, y) at *p* < 0.05. Abbreviations: aCO_2_, atmospheric CO_2_; eCO_2_, elevated CO_2_; N0, no N supply; N100, 100 mg N kg^−1^ DW soil.

The effects of CO_2_ and N supply on photosynthesis parameters during the booting stage of wheat was found to be diverse and differed between aCO_2_ and eCO_2_ conditions. Under aCO_2_, the application of N fertilizer significantly decreased the intercellular CO_2_ concentration (Ci) (Figure 2C), which suggested that higher N availability might reduce the internal CO_2_ concentration within the leaf, potentially due to increased photosynthetic activity or altered stomatal behavior. However, under eCO_2_, this effect was not observed, indicating that eCO_2_ might mitigate or alter the response of Ci to N supply. There was no significant change in the net photosynthesis rate (Pn) (Figure 2A), transpiration rate (E) (Figure 2B), and stomatal conductance (Gs) (Figure 2D) under both aCO_2_ and eCO_2_ with N100 supply. This suggested that while N supply could influence photosynthetic parameters, its impact on these specific metrics was not significant under these tested conditions.

All the measured parameters, including Ci, Pn, E, and Gs, were significantly greater under eCO_2_ than under aCO_2_ for both N0 and N100 treatments (Figure 2). This indicates that eCO_2_ generally enhanced wheat’s photosynthetic capacity, potentially due to the direct effect of higher CO_2_ levels on photosynthesis, known as the CO_2_ fertilization effect. This effect can increase the net photosynthetic rate by reducing photorespiration and increasing the efficiency of the Calvin cycle. Additionally, there was no significant change in the net photosynthetic rate of N0 under eCO_2_, suggesting that even without additional N supply, eCO_2_ can promote photosynthesis in wheat. Overall, these results indicated that eCO_2_ enhanced various aspects of photosynthesis in wheat during the booting stage, potentially leading to increased C assimilation and growth. The differential responses to N supply under aCO_2_ and eCO_2_ highlighted the importance of considering both CO_2_ levels and N availability when assessing plant photosynthetic performance and productivity.

### 2.3. Variation in Tissue N Concentrations

**Figure 3:**
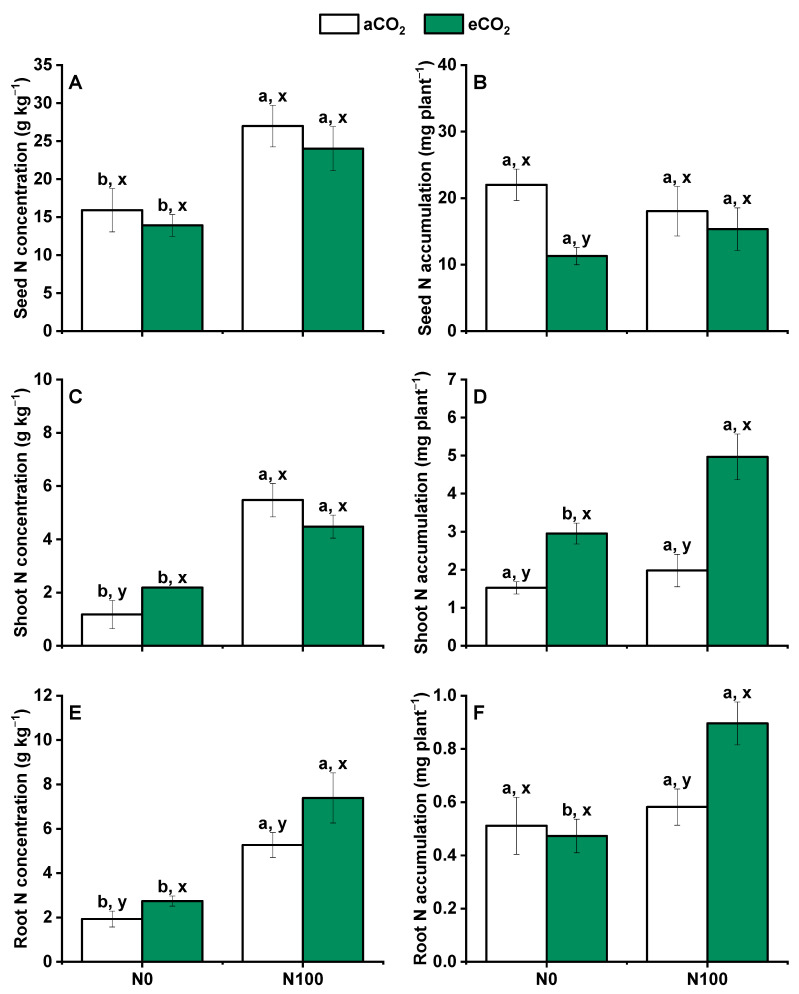
Effects of CO_2_ and N supply on tissue N concentrations and accumulations in seed (**A**,**D**), shoot (stem and leaf) (**B**,**E**) and root (**C**,**F**) of 5-month-old wheat at harvest. Data are means ± SE (*n* = 3). Lower-case letters above the bars indicate significant differences between N supplies for the same aCO_2_ treatment (a, b) or eCO_2_ treatment (**A**,**B**) and between CO_2_ treatments for the same N treatment (x, y) at *p* < 0.05. Abbreviations: aCO_2_, atmospheric CO_2_; eCO_2_, elevated CO_2_; N0, no N supply; N100, 100 mg N kg^−1^ DW soil.

Under aCO_2_ conditions, N100 resulted in a significant improvement in seed, shoot, and root N concentrations (Figure 3A,C,E), indicating that higher N availability enhanced N content in these tissues. However, this N supply did not significantly influence the seed, shoot, and root N accumulation (Figure 3B,D,F). These results indicate that while the concentration of N in plant tissues increased, the total amount of N that accumulated in these tissues did not change significantly. Under eCO_2_ conditions, N supply significantly increased N concentrations in seeds, shoots, and roots. This suggested that eCO_2_ enhanced the responsiveness of plants to N supply in terms of tissue N content. The level of N accumulation under N supply was significantly increased in shoots and roots but did not show a significant increase in seeds under eCO_2_. These results indicated that eCO_2_ might alter the distribution of N within the plant, favoring accumulation in certain tissues over others. Interestingly, shoot N concentrations for the N0 treatment were significantly greater under eCO_2_ than under aCO_2_, while they were similar in shoots for the N100 and also in seeds and roots for under N0 and N100, regardless of N fertilization. These findings suggested that eCO_2_ might influence N uptake or internal N redistribution even in the absence of additional N supply.

Regarding N accumulation in N0, seeds exhibited significantly decreased N accumulation, and shoots exhibited increased N accumulation under eCO_2_. Roots showed no significant change. This indicated a shift in N allocation under eCO_2_, potentially favoring shoot growth at the expense of seed N accumulation when the N supply is limited. The increasing trend of N accumulation in shoots and roots and the decreasing trend in seeds under eCO_2_ were further aggravated by N supply. This suggested that eCO_2_ and N supply interact to influence N distribution within the plant, potentially affecting crop yield and quality. Overall, the interactive effect of N supply and eCO_2_ on N concentrations was observed in both shoots and seeds (Figure 3), highlighting the complex interplay between CO_2_ levels and N availability in shaping plant N dynamics and potentially impacting agricultural productivity and sustainability.

### 2.4. Changes in Soil pH, Organic Matter, and Concentrations of NH_4_^+^ and NO_3_^−^

**Figure 4:**
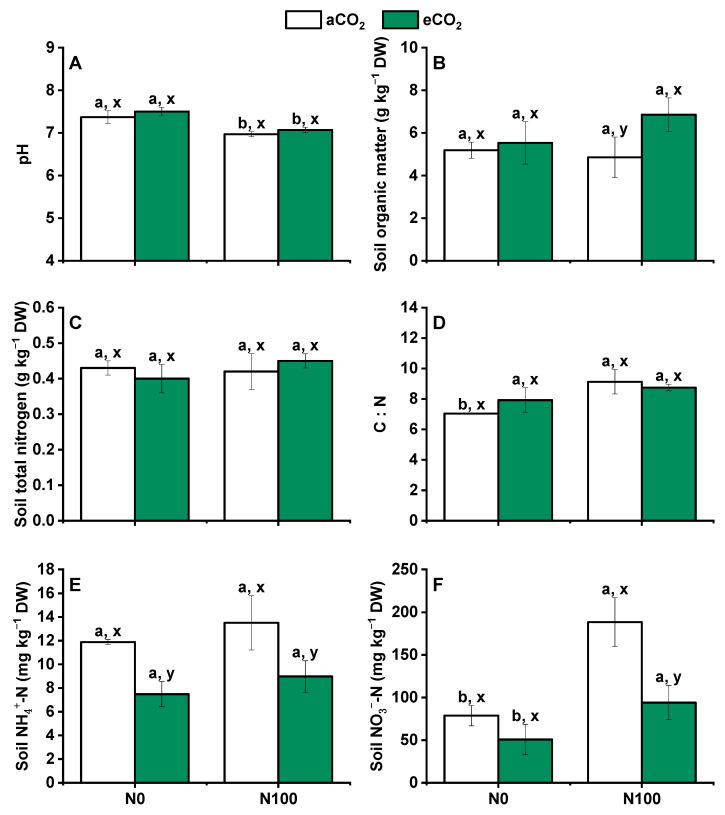
Effects of CO_2_ and N supply on (**A**) Soil pH; (**B**) Soil organic matter; (**C**) Soil total nitrogen; (**D**) C/N; (**E**) NH_4_^+^-N; (**F**) NO_3_^−^-N at harvest of 5-month-old wheat. Abbreviations: aCO_2_, atmospheric CO_2_; eCO_2_, elevated CO_2_; N0, no N supply; N100, 100 mg N kg^−1^ DW soil. Data are means ± SE (*n* = 3). Lower-case letters above the bars indicate significant differences between N supply for the same aCO_2_ treatment (a, b) or eCO_2_ treatment (**A**,**B**) and between CO_2_ concentrations for the same N treatment (x, y) at *p* < 0.05.

Under both aCO_2_ and eCO_2_, N supply led to a significant decrease in soil pH (Figure 4A). This was likely due to the acidifying effect of N fertilizers, which can release hydrogen ions into soil to decrease pH. Nitrogen supply did not result in changes in soil organic matter (Figure 4B), soil total N (Figure 4C), and soil NH_4_^+^-N concentrations (Figure 4E), but a significant increase in soil NO_3_^−^-N concentrations (Figure 4F) was noted, indicating that the N supply enhanced the availability of NO_3_^−^, a more mobile inorganic N form in soil. Under aCO_2_, the N supply also significantly increased the C-to-N (C/N) ratio. This suggested that while the N supply increased soil N, it might not proportionally increase soil organic matter, leading to a higher C/N ratio. Under both N0 and N100, the C/N ratio, soil NH_4_^+^-N, and soil NO_3_^−^-N were significantly lower under eCO_2_ than under aCO_2_ (Figure 4C–E). This indicated that eCO_2_ might influence the soil N cycle, potentially reducing the availability of both ammonium and NO_3_^−^ in soil. Contrastingly, both soil organic matter and total N exhibited significant increases under both N0 and N100, with the exception of soil organic matter under N0. This suggested that the N supply generally enhanced soil N and organic matter, but the response to eCO_2_ could vary depending on the level of N supply. Overall, these results indicated that the N supply decreased soil pH, while eCO_2_ decreased the C/N ratio, soil NH_4_^+^-N, and soil NO_3_^−^-N concentrations. These findings highlight the complex interactions among CO_2_ levels, N supply, and soil properties, which can influence soil fertility and plant nutrition.

### 2.5. Nitrogen Use Efficiency

Under N100 supply, eCO_2_ enhanced the agronomic efficiency of applied N (AE_N_) and physiological efficiency of applied N (PE_N_) compared to aCO_2_ conditions, as indicated in Table 1. This suggested that eCO_2_ promoted a more efficient use of applied N in terms of yield and biomass production per unit of N applied. However, eCO_2_ decreased the crop recovery efficiency of applied N (RE_N_) and partial factor productivity of applied N (PFP_N_) compared to aCO_2_ under N100 supply (Table 1). This indicated that while eCO_2_ improved the utilization efficiency of applied N within the plant, it might reduce the overall recovery of applied N by the crop and the productivity per unit of N applied in terms of total biomass or yield. These findings highlight the complex interactions between CO_2_ levels and N use efficiency in crops, with eCO_2_ potentially altering the balance among N uptake, utilization, and productivity.

### 2.6. Characters of Relationships between Soil Inorganic N and Plant N

**Figure 5:**
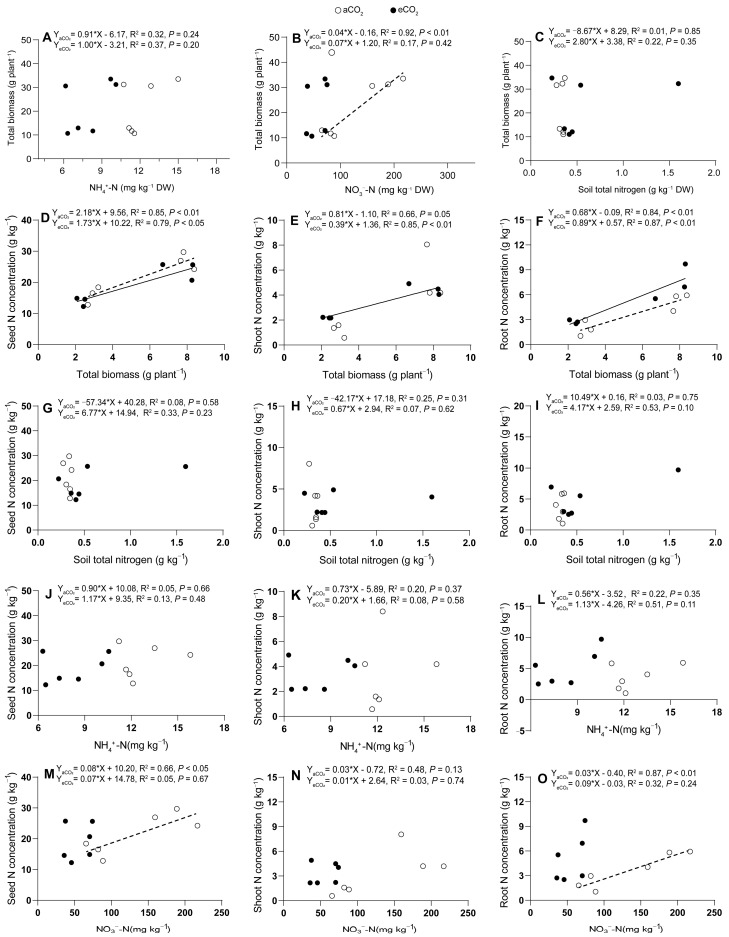
Relationships between soil NH_4_^+^-N (**A**), soil NO_3_^−^-N (**B**) or soil total nitrogen (**C**) and plant total biomass production (**A**–**C**); between tissue N concentrations in seed (**D**), shoot (**E**) or root (**F**) and plant total biomass production (**D**–**F**); between tissue N concentrations in seed (**G**), shoot (**H**) or root (**I**) and soil total nitrogen (**G**–**I**); between tissue N concentrations in seed (**J**), shoot (**K**) or root (**L**) and soil NH_4_^+^-N (**J**–**L**); between tissue N concentrations in seed (**M**), shoot (**N**) or root (**O**) and soil NO_3_^−^-N (**M**–**O**) in 5-month-old wheat grown under atmospheric CO_2_ (aCO_2_) and elevated CO_2_ (eCO_2_). Open and closed circles represent data under aCO_2_ and eCO_2_, respectively. Regressions are shown for aCO_2_ (dotted lines) and for eCO_2_ (solid lines) treatments, *n* = 6.

Wheat total biomass production exhibited varied correlations with soil and plant tissue N concentrations under different CO_2_ conditions. Under eCO_2_, wheat biomass production did not show significant correlations with soil NH_4_^+^-N (*p* = 0.20–0.24, Figure 5A), soil total N concentrations (*p* = 0.35–0.85, Figure 5C), or soil NO_3_^−^-N (*p* = 0.42, Figure 5B). Similarly, under aCO_2_, there was no significant correlation between wheat biomass production and shoot total N concentrations (*p* = 0.05, Figure 5E). However, under aCO_2_, wheat biomass production significantly positively correlated with soil NO_3_^−^-N (*p* < 0.01, Figure 5B), indicating a strong relationship between soil NO_3_^−^ availability and biomass production. Additionally, under eCO_2_, wheat biomass production showed a significant positive correlation with shoot N concentration (*p* < 0.05, Figure 5E), suggesting an enhanced role of shoot N in biomass production under eCO_2_. Furthermore, wheat biomass production significantly positively correlated with seed N concentration under both aCO_2_ and eCO_2_ (*P*_aCO_2__ < 0.01, *P*_eCO_2__ < 0.05, Figure 5D) and root N concentration under both aCO_2_ and eCO_2_ (*P*_aCO_2__ < 0.01, *P*_eCO_2__ < 0.05, Figure 5F), highlighting the importance of seed and root N accumulation in biomass production across different CO_2_ levels. Plant tissue total N concentrations, on the other hand, did not show significant correlations with soil total N concentrations (*p* = 0.10–0.75, Figure 5G–I), soil NH_4_^+^-N (*p* = 0.11–0.66, Figure 5J–L), or soil NO_3_^−^-N (*p* = 0.13–0.74, Figure 5M–O) except for seed and root N concentrations under aCO_2_, which significantly positively correlated with soil NO_3_^−^-N (*P*_seed_ < 0.05, *P*_root_ < 0.01). These suggest that while soil NO_3_^−^ availability is important for seed and root N accumulation under aCO_2_, the relationship between plant tissue N and soil N forms is complex and varies with CO_2_ levels and plant tissues.

**Figure 6:**
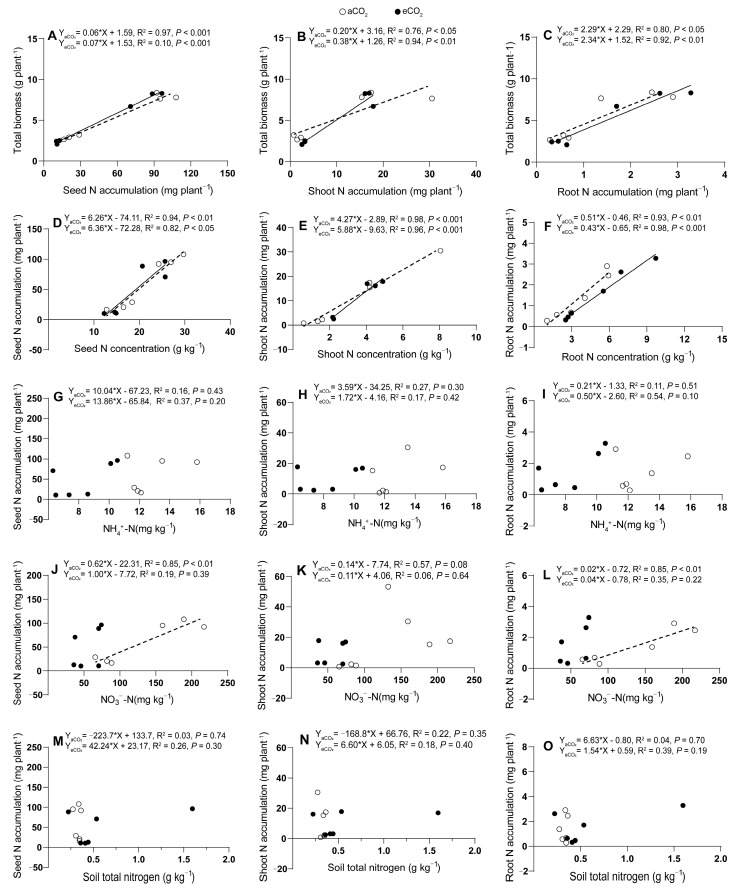
Relationships between tissue N accumulations in seed (**A**), shoot (**B**) or root (**C**) and plant total biomass production (**A**–**C**); between tissue N concentrations in seed (**D**), shoot (**E**) or root (**F**) and tissue N accumulations in seed (**D**), shoot (**E**) or root (**F**); between tissue N accumulations in seed (**G**), shoot (**H**) or root (**I**) and soil NH_4_^+^-N (**G**–**I**); between tissue N accumulations in seed (**J**), shoot (**K**) or root (**L**) and soil NO_3_^−^-N (**J**–**L**); between tissue N accumulations in seed (**M**), shoot (**N**) or root (**O**) and soil total nitrogen (**M**–**O**) in 5-month-old wheat grown under atmospheric CO_2_ (aCO_2_) and elevated CO_2_ (eCO_2_). Open and closed circles represent data under aCO_2_ and eCO_2_, respectively. Regressions are shown for aCO_2_ (dotted lines) and for eCO_2_ (solid lines) treatments, *n* = 6.

Wheat seed, shoot, and root N accumulation exhibited positive correlations with total wheat biomass production under both aCO_2_ and eCO_2_. Specifically, under aCO_2_, significant positive correlations were observed for seed (*p* < 0.001), shoot (*p* < 0.05), or root (*p* < 0.05) N accumulation and total biomass production (Figure 6A–C). Under eCO_2_, these correlations became even stronger, with seed (*p* < 0.001), shoot (*p* < 0.01), and root (*p* < 0.01) N accumulation showing highly significant positive relationships with total biomass (Figure 6A–C). The eCO_2_ enhanced relationship between biomass production and shoot N accumulation suggested a more efficient utilization of N in biomass production under eCO_2_. Plant tissue total N concentrations and N accumulations were found to correlate significantly positively under both aCO_2_ and eCO_2_. Under aCO_2_, significantly positive correlations were observed for seed (*p* < 0.01), shoot (*p* < 0.001), and root (*p* < 0.01) N concentrations with total biomass (Figure 6D–F). Under eCO_2_, these correlations persisted, with seed (*p* < 0.05), shoot (*p* < 0.001), and root (*p* < 0.001) N concentrations showing significantly positive relationships with total biomass (Figure 6D–F). However, wheat tissue N accumulation did not show significant correlations with soil NH_4_^+^-N (*p* = 0.10–0.51, Figure 6G–I), soil total N concentrations (*p* = 0.19–0.74, Figure 6M–O), or soil NO_3_^−^-N (*p* = 0.08–0.64, Figure 6J–L) under both aCO_2_ and eCO_2_. Soil NO_3_^−^-N was found to correlate significantly and positively with seed and root N accumulation (*P*_seed_ < 0.01, *P*_root_ < 0.01, Figure 6J,L), indicating a specific relationship between soil NO_3_^−^-N availability and N accumulation in these plant tissues.

### 2.7. Soil Microbial Community Composition and Species Abundance

The differences in the soil microbial community between different treatments were assessed using Illumina MiSeq sequencing of the V3–V4 region of bacterial 16S rRNA gene. These Illumina MiSeq sequencing assays were performed by followed by the instructions from the facility of an Il-559 lumina, San Diego, CA, USA. A total of 516,804 valid sequences were obtained from the soil sample, with an average of 43,067 individual sample sequence counts per sample.

**Figure 7:**
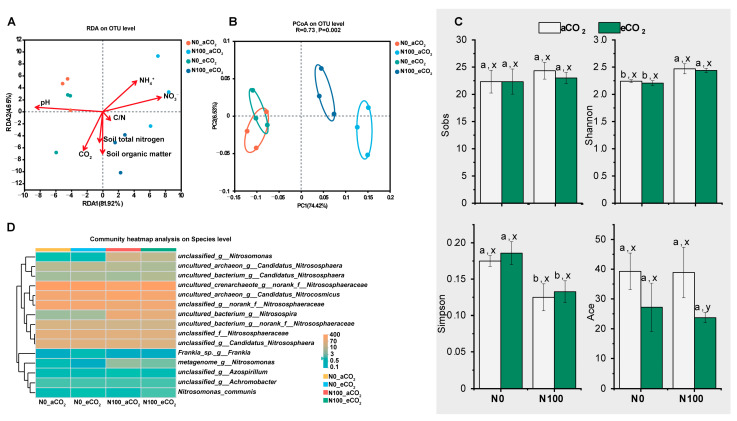
Transition of soil microbes under different CO_2_ levels and N supply. (**A**) Redundancy analysis (RDA) plot showing the relationship between environmental factors and microbial community structure at the OTU level; (**B**) Principal coordinate analysis (PCoA) of microbial community composition at the OTU level; (**C**) Sobs, Shannon, Simpson, and Ace microbial diversity indexes at the OTU level. Data are means ± SE (*n* = 3). Lower-case letters above the bars indicate significant differences between different N supply treatments for the same CO_2_ treatment (a, b) and between different CO_2_ concentrations for the same N treatment (x, y) at *p* < 0.05; (**D**) Heat map shows the relative abundance of the microbial community at the species level. Abbreviations: aCO_2_, atmospheric CO_2_; eCO_2_, elevated CO_2_; N0, no N supply; N100, 100 mg N kg^−1^ DW soil.

Alpha diversity is an ecological metric that assesses the variety and even distribution of taxonomic groups within a single sample [32]. Elevated CO_2_ did not significantly affect the Sobs index, Shannon index, or Simpson index under both N0 and N100, while the Ace index had a decreasing trend. Under both aCO_2_ and eCO_2_, N100 significantly increased the Shannon index but decreased the Simpson index (Figure 7C). N100 significantly influenced N cycling microorganisms (*p* = 0.002), while the influence of eCO_2_ on communities was higher under N100 than under N0 (Figure 7B). We performed redundancy analysis (RDA) to show the correlation between samples under different treatments, environmental factors, and soil N cycling microorganisms. The RDA results (RDA1 = 81.92%, RDA2 = 4.65%) showed that soil pH was the most influential factor on soil N cycling microorganisms followed by NH_4_^+^-N and NO_3_^−^-N (Figure 7A). Under both aCO_2_ and eCO_2_, the N supply notably increased the relative abundance of unclassified_g_*Nitrosomonas* and uncultured_bacterium_g_*Nitrosospira* at the species level. eCO_2_ treatment notably decreased metagenome_g_*Nitrosomonas* and increased *Frankia*_sp._g_*Frankia* under N0 (Figure 7D).

**Figure 8:**
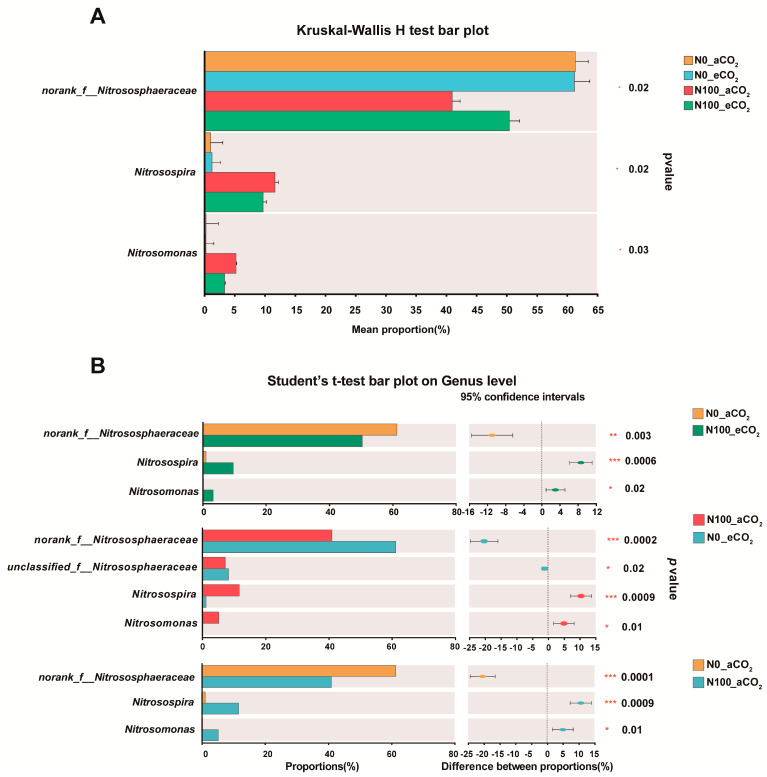
Kruskal-Wallis H test (**A**) and Student’s *t*-test (**B**) of the relative abundance of the three most abundant genera. Extended error bar plot showing the most abundant genera that had significant differences between the treatments. Positive differences in mean relative abundance indicate a genus overrepresented in the soil of the treatment, while negative differences indicate greater abundance in the soil of the treatment. Abbreviations: aCO_2_, atmospheric CO_2_; eCO_2_, elevated CO_2_; N0, no N supply; N100, 100 mg N kg^−1^ DW soil; *, **, *** were considered statistically significant for *p* < 0.05, *p* < 0.01, *p* < 0.001, respectively.

Significant differences among four treatments were observed in the relative abundance of soil N cycling microorganisms at the genus level of the 3 most abundant genera (Figure 8A). These 3 genera were dominating the communities for N cycling in soil from all treatments. *Nitrososphaeraceae* was the main microbial genus present at N0 under both aCO_2_ and eCO_2_ (*p* < 0.001–0.01). *Nitrosospira* and *Nitrosomonas* in the soil under N100 treatment were more abundant compared to N0 under both aCO_2_ and eCO_2_ (*p* < 0.001–0.05) (Figure 8B). These results meant that the N supply was the main factor influencing soil microbial communities.

## 3. Discussion

### 3.1. Nitrogen Supply Generally Rebalanced the Effects of eCO_2_ on Wheat Growth

Elevated CO_2_ (eCO_2_) did not significantly increase wheat biomass production; in fact, it somewhat reduced the growth biomass under N0 (Figure 1B–E). This result was in a disagreement with results from other studies [5,9,33] because a good normal biomass production needs an element match-up of C and N stoichiometry [34] and also highly depends on cultivars as two out five wheat cultivars had a lower shoot dry weight production under 800 ppm CO_2_ than under 400 ppm CO_2_ [9]. Compared to aCO_2_ (400 ppm), an NH_4_^+^-tolerant pea (*Pisum sativum* L. cv. snap pea) plant under eCO_2_ (800 ppm) had to cope with an unbalanced C-to-N ratio due to a limited C sink strength and/or constrains in leaf N content in order to promote photosynthetic efficiency and C allocation into sugars (glucose and sucrose) under moderate (2.5 mM) or high (10 mM) NH_4_^+^ supplement [35]. Moreover, a meta-analysis of 22 studies showed that an enhanced aboveground biomass production and grain yield was only observed when 18 barley genotypes were cultivated under a combination of eCO_2_ (651–720 ppm) and a higher N rate (151–200 kg ha^−1^) [36]. In addition, a review on studies between 1986 and 2022 (*n* = 20) showed that strawberry yield was also decreased under 1588 ± 582 CO_2_ ppm than under aCO_2_ [37]. Nevertheless, the N supply and CO_2_ treatment did not significantly influence the harvest index (Figure 1F). In general, the effect of CO_2_ fertilization on photosynthesis had been significantly reduced globally during the last four decades (1982 to 2015) [38], although the study’s key conclusion was not robust mainly due to negligence regarding the role of the photosynthetic enzyme ribulose-1,5-bisphosphate carboxylase-oxygenase [39].

When the environmental CO_2_ concentration increased from 372 ppm to 605 ppm, the response for wheat yield indicated that at about 600 ppm CO_2_, the stimulation of yield tended to stabilize, suggesting the existence of a plateau phase, beyond which the yield might not be significantly increased [40]. Furthermore, the response of wheat to the increase in CO_2_ concentration is significantly related to the productivity of the growth site [41]. In systems with a lower productivity, the relative stimulation effect of CO_2_ on yield was stronger [42]. Wheat biomass production was significantly increased by N100 supply under both atmospheric CO_2_ (aCO_2_) and eCO_2_ (Figure 1B–E), which was consistent with results from other studies [43,44]. This suggests that N generally boosts wheat biomass by supporting essential physiological processes, whereas eCO_2_ decreases biomass by disrupting nutrient balance and water relations or limitations. In the latter case, our results indeed showed that eCO_2_ significantly decreased the water use efficiency at the leaf scale (WUEi) under N0 (Figure S2). As a result, the fertilization effect of eCO_2_ on wheat biomass production can only be achieved when N and/or water supplementation are adequate. This illustrates the complex interplay between these factors in plant growth.

### 3.2. Elevated CO_2_ Changed the Decreasing Trend of N Supply on Photosynthesis Parameters

The effects of N fertilizer and CO_2_ concentration on the photosynthetic parameters of wheat were different. The stomatal conductance and net photosynthetic rate were lower at N100 than at N0 under aCO_2_. In addition, the intercellular CO_2_ concentration was significantly decreased at N100 under aCO_2_, but the photosynthesis parameters under eCO_2_ were not significantly affected by N supply during the booting stage (Figure 2). Nitrogen is essential for plant photosynthesis because its availability significantly influences the efficiency of the photosynthetic process and, by extension, the overall growth and performance of plants [45]. A study performed by Wu et al. also showed that N application had significant decrease on the intercellular CO_2_ concentration of wheat at the booting stage [46]. Applying excessive N fertilizer may lead to an over-absorption of N by plants, which can increase the chlorophyll content in wheat, enhance the rate of photosynthesis, and thus increase the rate of CO_2_ absorption, resulting in a reduction of leaf intercellular CO_2_ concentrations [46,47,48]. Elevated CO_2_ significantly increased the photosynthetic parameters under both N0 and N100 (Figure 2). A study measured photosynthesis in winter wheat and rice under different CO_2_ levels and also found that eCO_2_ generally increased net photosynthesis by 10–40% at different development stages [49,50]. The increase in CO_2_ concentration to some extent compensates for the restrictive effect of N fertilizer application on the formation during the booting stage.

### 3.3. Nitrogen Supply Mitigated the Reduction of Seed N Accumulation with eCO_2_ Treatment

Elevated CO_2_ increased N concentrations in both shoots and roots of wheat but decreased the seed N concentration under N0 (Figure 3A,C,E, Figure 5J,M). N100 enhanced such increasing trends in seed and root N concentrations, while shoot N concentrations were decreased under eCO_2_ (Figure 3A,C,E). These results were similar to Andrea et al.’s study demonstrating that eCO_2_ reduced the N content and nutritional quality of wheat and other crops [51,52]. Elevated CO_2_ significantly decreased seed N accumulation under N0, while this trend was stopped under N100 (Figure 3B). These results might explain why eCO_2_ and increased N fertilizer significantly increased the number of tillers (Figure S1) and aboveground dry biomass of wheat (Figure 1) but reduced the leaf chlorophyll content (as a proxy for plant N content) [53]. However, of note, N accumulation in shoots and roots was much higher at eCO_2_ than at aCO_2_ under N100 (Figure 3D,F). Studies have focused on the regulation of N allocation in plants by N application rates. During the period from flowering to maturity, the N translocation in individual plant aboveground organs decreased with the downward spatial position [54]. Increased N application could effectively enhance the absorption of N, delaying leaf senescence and death (Figure 5O and Figure 6L). It is hence important to fertilize at the proper stage of wheat.

### 3.4. Elevated CO_2_ Decreased Active Nitrogen under Both N0 and N100

The pH decreased because of nitrification ($2NH_{4}^{+}+3O_{2}\to 2NO_{3}^{-}+4H^{+}+2H_{2}O$) under both aCO_2_ and eCO_2_, which is important for plant growth [55] (Figure 4A). The concentrations of NH_4_^+^ and NO_3_^−^ under eCO_2_ were significantly decreased under both N0 and N100, while soil total N and C/N under aCO_2_ and eCO_2_ did not significantly change under both N0 and N100 (Figure 4C,E,F). The increase in CO_2_ concentration could increase the biomass in the upper part of the crop, thereby increasing the crop’s demand for N [56]. The average soil total N value is 0.9 g kg^−1^ [57], and it was barren compared to the soil used in this study (soil total N = 0.53 g kg^−1^). Studies have shown that an increased CO_2_ concentration can promote the growth and development of crop root systems, expand the distribution of root systems in soil, and increase the absorption of N [58]. Because the concentration of CO_2_ is much higher (about 10–50 times) in soil than in the atmosphere, the increase in the CO_2_ concentration in the atmosphere has almost no direct impact on soil microorganisms [59]. Instead, it has an indirect effect on soil microorganisms by affecting the root secretions and falling objects of the crop, further affecting soil N conversion [60].

### 3.5. Elevated CO_2_ Increased N Use Efficiency for Plant Growth but Not for Yield Production under N100

Research has shown that N use efficiency is a critical factor in agricultural production and environmental sustainability [61]. The concept of N use efficiency is defined as the flux ratio between plant dry matter production and N uptake, which is essential for understanding plant growth strategies and ecosystem productivity [62]. The physiological efficiency of applied N (PE_N_) indicates the amount of biomass produced per unit of N absorbed by the plant [63], while the partial factor productivity of applied N (PFP_N_) reflects the yield increase per unit of N input [64], showing the marginal return on N use [65]. The crop recovery efficiency of applied N (RE_N_, [66]) is an indicator of how much of the N input is effectively utilized by the crop, while AE_N_ (agronomic efficiency of applied N) is often used to evaluate the effectiveness of N fertilizers [67]. Elevated CO_2_ decreased RE_N_ and PFP_N_ and increased AE_N_ and PE_N_ under N100 in this study (Table 1). These results were supported by the N concentrations and accumulations (Figure 1, Figure 3, Figure 5 and Figure 6). This could mean an increase in the growth rate and overall biomass of plants in eCO_2_ environments, even if the plants are not grown to harvest grains or produce high yields. Elevated CO_2_ generally results in increased grain production but lower grain quality, particularly with regard to N concentration and protein content, which can lead to increased “hidden hunger” problems [68]. The free amino acid and protein contents of cereals grown under eCO_2_ conditions are expected to decrease significantly [42]. The availability of N limited the effect of CO_2_ fertilization; that is, eCO_2_ increased plant biomass more significantly when the supply of N was sufficient [52]. Total biomass is related positively to seed, shoot, and root N concentration and accumulation under both aCO_2_ and eCO_2_ (only eCO_2_ for shoot N concentration) (Figure 5D–F and Figure 6A–C). Soil NO_3_^−^-N is related positively to seed and root N concentration and accumulation under aCO_2_ (Figure 5M,O). These results were similar to common eCO_2_ results indicating an increase of grain yield but a decrease of grain quality, particularly in N concentration and protein content [42,68].

### 3.6. Influence of eCO_2_ and N100 on the Relative Abundance and Structure of N Cycling Microorganisms

Nitrogen supply led to the decreasing pH, which is the most important factor for microbial community composition in this study (Figure 4A and Figure 7A). Soil acidification is a critically topic soil issue, and it has also been demonstrated that soil acidification significantly influenced plant diversity, species richness, and the occurrence of species [69]. Previous research indicates that soil acidification can result from N deposition, acid rain, and continuous cropping practice [70,71,72,73]. Nutrient-induced changes in soil pH are a primary driver controlling diversity–function relationships [74]. Although eCO_2_ significantly influenced plant production, it did not significantly affect soil N cycling microorganisms (Figure 1 and Figure 7B,C). A study has shown that eCO_2_ had no significant effect on nitrification, while AOA communities are more responsive to elevated temperature than AOB communities [75]. N100 significantly increased the richness and evenness of the community and the relative abundance of *Nitrosomonas* and *Nitrosospira* (Figure 7B–D). *Nitrososphaeraceae* was significantly higher at N0 than at N100 under both aCO_2_ and eCO_2_, while contrasting results were obtained for *Nitrosomonas* and *Nitrosospira* (Figure 8B). Different N requirements are noted between archaea and bacteria involved in N oxidation; for example, the requirements for *Nitrososphaeraceae* (archaea) versus *Nitrosomonas* and *Nitrosospira* (bacteria) are influenced by their distinct ecological roles, metabolic capacities, and evolutionary adaptations [76]. AOA communities often thrive in environments with low ammonia concentrations and can tolerate a wider range of temperatures and pH levels, while AOB communities typically prefer higher ammonia concentrations and may be more sensitive to extreme environmental conditions [77]. These may be the reason why AOA and AOB communities differentiate beneath soil niches under different N supplies and eCO_2_ levels.

## 4. Materials and Methods

### 4.1. Description of the Experiment Site

The experiment site is located in the National Monitoring Base of Purple Soil Fertility and Fertilizer Effect (29°48′ N, 106°24′ E, 266.3 m above sea level) on the Beibei campus of Southwest University, Chongqing, China, which is located within the purple hilly region with a subtropical monsoon climate. Over the past three decades, the mean annual sunshine was 1276.7 h, the mean annual precipitation was 1145.5 mm, and the mean annual temperature was 18.4 °C. During the experiment period, the atmospheric CO_2_ (aCO_2_) concentration was ~415 ppm at this experiment site. The soil is classified as a purple soil (Eutric Regosol, FAO Soil Classification System), which has evolved from purple mud and shale of the Jurassic Shaximiao Formation [78] and exhibits the following basic chemical properties: pH 7.4 (1:2.5 *W*/*V*, soil/water), 9.00 g kg^−1^ organic matter, 0.53 g kg^−1^ total N, 7.81 mg kg^−1^ NH_4_^+^, and 16.47 mg kg^−1^ NO_3_^−^.

### 4.2. Design and Description of Custom-Built Chambers

The experiment was carried out in 6 identical enclosed gas chambers (length × width × height = 1.5 m × 1.0 m × 2.5 m), which were made of a 10 mm thickness steel frame covered with transparent glass (90% light transmission rate) (Yutao Glass Company, Chongqing, China). The gas flow solenoid valve (AirTAC (China)Co., Ltd., Ningbo, China) was connected to the metal cylinder containing pure CO_2_ as the gas source. Each chamber had two air pumps (suction and intake), and the excess CO_2_ and water vapor were balanced with 1 M NaOH solution and anhydrous CaCl_2_ in the chamber, respectively. A hanging air conditioner (Gree, Zhuhai Gree company, Zhuhai, China) was installed on the top of the chamber to regulate the air temperature. An atmospheric light, temperature, and humidity sensor (Jingxun Electronic Technology, Weihai, China) and CO_2_ concentration detector (infrared CO_2_ sensor module B-530, ELT SENSOR Corp, Bucheon-si, Republic of Korea) were installed at the middle of the chamber. All these devices were deployed using fully automatic control device (DSS-QZD, Qingdao Shengsen Numerical Control Technology Institute, Qingdao, China). The whole system can automatically control the temperature, humidity, and CO_2_ concentration inside and outside the glass chamber and ensure that the CO_2_ concentration was maintained as required by the experiment in the chamber [30].

### 4.3. Designs of Experiment and Preparation of Materials

Based on the field-detected CO_2_ concentration, we set up two CO_2_ concentration treatments: (1) atmospheric CO_2_ (aCO_2_, 410 ppm during daytime/460 ppm at night) and (2) eCO_2_ (eCO_2_, 550 ppm during daytime/610 ppm at night). The time of day and night for CO_2_ treatment varied with the local sunrise and sunset and also with seasons.

Wheat seeds (*T. aestivum* cv. Yunmai) were sterilized with a 6% (*v*/*v*) hydrogen peroxide and germinated on sterile filter paper [79]. Germinated wheat seeds were sown in plastic pots (diameter = 22 cm, height = 20 cm, each containing 5 kg soil), and 4 uniform seedlings were grown inside the growth chambers until they reached the harvest stage (5 months old).

Along with the addition of P (100 mg P kg^−1^ DW soil, Ca(H_2_PO_4_)_2_) and K (126 mg K kg^−1^ DW soil, K_2_SO_4_), two N fertilization treatments were also applied as follows: (1) no N supply (N0) and (2) 100 mg N kg^−1^ DW soil (N100). As a result, the experiment had four treatments (two CO_2_ levels and two N fertilization rates) for a total of 12 pots (each treatment had three pots or replicates). All fertilizers were thoroughly mixed with the soil before wheat planting. The potting preparation, row spacing, N supply, and irrigation followed common cultivation practices in the local area, and no pesticides or fungicides were used. To ensure a similar environment for all plants, the potted plants in the growth chamber were rotated once per week. Adequate irrigation was provided to maintain soil moisture at ~ 70 ± 5% of field capacity.

### 4.4. Measurement of Photosynthetic Parameters

During the grain booting stage of wheat, photosynthetic parameters were measured. Wheat plants with similar growth were selected, and the flag leaves of their main stem were used for measuring photosynthetic parameters. These measurements were conducted between 8:30 to 11:30 a.m. in sunny morning of 9 April 2019 using a Li-6400XT portable photosynthesis system (Li-Cor Inc., Lincoln, NE, USA) equipped with an internal red-blue light source. The light intensity was set to 1000 μmol m^−^^2^ s^−1^. The CO_2_ concentration in the reference chamber was set to 410 μmol mol^−1^ for the N0 and N100 treatments under aCO_2_ and 550 μmol mol^−1^ for the N0 and N100 treatments under eCO_2_. The net photosynthetic rate (Pn), stomatal conductance (Gs), intercellular CO_2_ concentration (Ci), and transpiration rate (E) were recorded, respectively.

The formula for calculating instantaneous water use efficiency at the leaf scale (WUEi) is given by [80,81]:

$${\mathrm{WUE}}_{i}=\frac{\mathrm{Pn}}{E}$$

Here,

- Pn is the leaf net photosynthetic rate (μmol CO_2_ m^−2^ s^−1^);
- E is the transpiration rate (μmol H_2_O m^−2^ s^−1^); and
- WUE_i_ is the instantaneous water use efficiency at the leaf scale (μmol CO_2_ μmol^−1^ H_2_O).

### 4.5. Preparation of Plant and Soil Samples

Plant and soil samples were collected at wheat harvest (5 months old) as depicted in Figure S3 by individuals wearing disposable gloves to avoid contamination. Plant height measurements were conducted using a tape measure from the ground to the top of the spike (excluding awns) and recorded in centimeters immediately prior to destructive sampling. Plants were further divided into roots, stems, leaves, and ears. Plant samples were dried in an oven at 70 °C for 72 h. The ears were threshed, and the number of grains per plant was also documented. The dry weight of seed, shoot, and root biomass production was recorded. Harvest index was determined using the established formula as previously described in the literature [82,83]:

$$H\mathrm{arvest}\;\mathrm{index}=\frac{\mathrm{Seed}\;\mathrm{production}}{\mathrm{Shoot}\;\mathrm{biomass}\;\mathrm{production}}$$

A total of 10 soil cores from different locations within the same pot were collected to create a composite sample for minimizing spatial variability. Any plant material or debris was removed from soil samples. The collected soil samples were packed in sterile Ziplock bags, transported to the laboratory in a portable refrigerator (−18 °C), and stored at −80 °C for soil DNA extraction. A portion of soil samples were ground through 2 mm and 0.25 mm sieves and air-dried for soil physical and chemical properties analysis.

### 4.6. Determination of Plant and Soil Chemical Characters

Using a LE438 composite electrode meter (Mettler Toledo Instrument Co., Ltd., Shanghai, China), soil pH was determined with a soil to water ratio of 1:2.5 (*W*/*V*). Determination of soil organic matter was performed using the K_2_Cr_2_O_7_ external heating method, and soil total N was assessed using the Kjeldahl method. Soil soluble inorganic N (NH_4_^+^-N and NO_3_^−^-N) was measured using colorimetric methods with a spectrometer (UV-1800, AOE Instruments, Shanghai, China) at 625 nm (only the same amount of reagent but no soil leaching solution at 625 nm as a blank control) [84] and 275 nm (deionized water at 220 nm as blank control) [85], respectively. Plant total N was determined using the H_2_SO_4_-H_2_O_2_ digestion and distillation method, which involved boiling the test solution with a mixture of sulfuric acid and hydrogen peroxide. All the parameters were determined according to relevant methodologies [86].

### 4.7. Calculations of Nitrogen Use Efficiency

RE_N_—Crop recovery efficiency of applied N (g increase in N uptake per g N applied):

RE_N_ (%) = (U_N_ − U_0_)/F_N_

PE_N_—Physiological efficiency of applied N (g yield increase per g increase in N uptake from fertilizer):

PE_N_ (g g^−1^) = (Y_N_ − Y_0_)/(U_N_ − U_0_)

PFP_N_—Partial factor productivity of applied N (often simply called N use efficiency or NUE) (g harvest product per g N applied):

PFP_N_ (g g^−1^) = Y_N_/F_N_

AE_N_—Agronomic efficiency of applied N (g yield increase per g N applied):

AE_N_ (g g^−1^) = (Y_N_ − Y_0_)/F_N_

The meanings of these short terms were as follows: F_N_—amount of (fertilizer) N applied (g kg^−1^ DW); Y_N_—crop yield with applied N (g kg^−1^ DW); Y_0_—crop yield (g kg^−1^ DW) in a control treatment with no N; U_N_—total plant N uptake in aboveground biomass at maturity or harvest (g kg^−1^ DW) in a plot that received N; U_0_—the total N uptake in aboveground biomass at maturity or harvest (g kg^−1^ DW) in a place that received no N.

### 4.8. Analysis of Soil Bacterial and Archaeal Community Based on Illumina Sequencing

Total DNA of soil microorganisms was extracted from 2 mL sludge using a FastDNA^®^ SPIN Kit for Soil (MP Biomedicals, LLC, Irvine, CA, USA). The specific operation was strictly performed in accordance with the kit’s instructions. The extracted total microbial DNA was stored in a refrigerator at −20 °C for future assays. The extracted genomic DNA was assessed using 1% agarose gel electrophoresis (Bio-Rad Inc., Hercules, CA, USA) and a NanoDrop-2000 Spectrophotometer (NanoDrop Technologies Inc., Wilmington, DE, USA). Three replicates were extracted from each composite soil sample, and the extracted DNA was pooled together. Each treatment had three composite DNA samples. The bacterial community composition of rhizosphere soil was analyzed using high-throughput amplification sequencing. A forward primer 515FmodF (GTGYCAGCMGCCGCGGTAA) and a reverse primer 806RmodR (GGACTACNVGGGTWTCTAAT) were used for bacterial and archaeal 16S rRNA gene PCR amplification of the V4 region and then sequenced by following the assay instructions of Illumina MiSeq sequencing technology (Il-559 Illumina, San Diego, CA, USA). The operational taxonomic units (OTUs) were classified by Usearch (v7.1) with a 97% sequence similarity threshold. The microbial community structure and relative abundance were obtained by OTUs with an online platform, namely, the Majorbio Cloud (https://cloud.majorbio.com/ (accessed on 20 June 2024)).

### 4.9. Statistical Analysis

SPSS 19.0 (SPSS Inc., Chicago, IL, USA) was used to analyze differences between different treatments using one-way ANOVA. Data (means ± SE, *n* = 3) were compared using the Duncan’s multiple range test at *p* < 0.05 level. GraphPad Prism (GraphPad Software 8.0.2) was used to analyze characters of relationships. Alpha diversity analyses were completed utilizing the QIIME2 platform, followed by a visualization of all the results with graphical representations generated using mothur (version v.1.30.2 https://mothur.org/wiki/calculators/ (accessed on 21 June 2024) [87]. Variations in the relative abundances of bacterial species were described in a heat map that was generated using the vegan package in the 3.3.1 R version. Principal coordinate analysis (PCoA) was achieved based on the Bray-Curtis distance matrix derived from the OTU information of each sample using R version 3.3.1 [88]. Redundancy analysis (RDA) was performed to clarify the relationships between sample distributions and environmental factors [89]. First, detrended correspondence analysis (DCA) was applied to the species-sample data matrix, derived from 97% OTU similarity, to determine the gradient length. Based on the DCA results, the bioenv function was then used to assess the maximum Pearson correlation coefficient between environmental factors and community distributions for identifying a subset of significantly environmental factors. The species distribution table and the complete set of environmental factors or the identified subset were then subjected to RDA. The significance of the RDA results was evaluated using a permutation test analogous to ANOVA, implemented using the vegan package of R version 3.3.1. This package also facilitated the RDA analysis and graphical representation. The Kruskal–Wallis H test and the Student’s t test were performed using the stats package in R version 3.3.1. Briefly, the Kruskal–Wallis H test, a non-parametric method, was employed to assess differences among multiple groups of samples, while the Student’s t test for equal variances was utilized to evaluate whether the means of two sample groups with homoscedasticity (equal variances) were identical. These analyses were performed to determine significant differences in species distribution between the groups and to adjust the *p* values using appropriate multiple correction methods.

## 5. Conclusions

In this study, we examined the impact of elevated CO_2_ levels (eCO_2_) and N (N) supply on wheat growth, photosynthesis, and N accumulation in plant tissues as well as soil microbial community dynamics. Our findings indicate that the N supply can counteract the growth suppression effect caused by eCO_2_ by significantly reducing intercellular CO_2_ concentrations and enhancing photosynthesis parameters. The application of N increased the concentration of N in the seeds, shoots, and roots of wheat, with biomass production under eCO_2_ further contributing to enhanced N accumulation in these plant parts. This suggests that a higher requirement for N under eCO_2_ conditions improves N utilization and absorption efficiency in wheat. Additionally, N supply was found to significantly increase the richness and evenness of the microbial community, indicating an impact on soil biodiversity. Our study also shed light on the responses of specific nitrification-related microbial taxa, such as *Nitrososphaeraceae*, *Nitrosospira*, and *Nitrosomonas*, to N supply under both atmospheric CO_2_ (aCO_2_) and eCO_2_ conditions. These microbes exhibited differential responses, with N supply essentially dividing the microbial communities into two distinct groups under both CO_2_ conditions. The findings underscore the critical role of increased N supply in enhancing seed N accumulation, facilitating the activity of nitrification-related microorganisms, and potentially improving wheat growth and soil health. This research highlights the importance of adjusting N fertilization strategies to mitigate the challenges posed by elevated CO_2_ levels, thus contributing to the sustainable improvement of soil fertility and plant productivity in the context of climate change.

## Figures and Tables

**Table 1:** Agronomic indices of N use efficiency.

	RE_N_ (%)	AE_N_ (g g^−1^)	PE_N_ (g g^−1^)	PFP_N_ (g g^−1^)
aCO_2_	37.35 ± 2.27 x	8.89 ± 0.89 x	23.89 ± 3.1 x	0.15 ± 0.01 x
eCO_2_	34.24 ± 5.44 x	10.84 ± 3.16 x	31.3 ± 6.02 x	0.14 ± 0.03 x

Abbreviations: aCO_2_, ambient CO_2_; eCO_2_, elevated CO_2_. Data are means ± SE (*n* = 3). Lower-case letters above the bars indicate a significant difference between CO_2_ concentrations in the same N treatment (x, y) at *p* < 0.05.

## Data Availability

Data are contained within the article. The data presented in this study are openly available in NCBI Sequence Read Archive (SRA) database at https://www.ncbi.nlm.nih.gov (accessed on 24 July 2024), reference number PRJNA1139369.

## Supplementary Materials

The following supporting information can be downloaded at: https://www.mdpi.com/article/10.3390/plants13172345/s1. Figure S1. Effects of CO_2_ and N supply on tillers of 5-month-old wheat plants at harvest. Abbreviations: aCO_2_, atmospheric CO_2_; eCO_2_, elevated CO_2_; N0, no N supply; N100, 100 mg N kg^−1^ DW soil. Data are means ± SE (*n* = 3). Lower-case letters above the bars indicate significant differences between N supply for the same CO_2_ treatment (a, b) and between CO_2_ concentrations for the same N treatment (x, y) at *p* < 0.05. Figure S2. Effects of CO_2_ and N supply on WUEi of wheat plants at the booting stage. Abbreviations: aCO_2_, atmospheric CO_2_; eCO_2_, elevated CO_2_; N0, no N supply; N100, 100 mg N kg^−1^ DW soil; WUEi, the instantaneous water use efficiency at the leaf scale (μmol CO_2_ μmol^−1^ H_2_O). Data are means ± SE (*n* = 3). Lower-case letters above the bars indicate significant differences between N supply for the same CO_2_ treatment (a, b) and between CO_2_ concentrations for the same N treatment (x, y) at *p* < 0.05. Figure S3. Effects of different CO_2_ concentrations and N supply on the growth status of 5-month-old wheat plants at harvest. Abbreviations: aCO_2_, atmospheric CO_2_; eCO_2_, elevated CO_2_; N0, no N supply; N100, 100 mg N kg^−1^ DW soil.
