# Supplementary material for: Elevated CO2 and Nitrogen Supply Boost N Use Efficiency and Wheat (T. aestivum cv. Yunmai) Growth and Differentiate Soil Microbial Communities Related to Ammonia Oxidization
Source: Plants (Basel). 2024 Aug 23;13(17):2345. doi: 10.3390/plants13172345 (PMC11397272; doi:10.3390/plants13172345)
Supplement: Supplementary file 1 [file plants-13-02345-s001.zip › plants-3148038-supplementary.pdf]

Supplementary material

Elevated CO_2 and Nitrogen Supply Boost N Use Efficiency and Wheat Growth, and Differentiate Soil Microbial Communities Related to Ammonia-oxidization

Xingshui Dong [1], Hui Lin [2], Feng Wang [2], Songmei Shi [1], Sharifullah Sharifi [1], Shuai Wang [1], Junwei Ma [2*] and Xinhua He [1,3,4*]

[1] National Base of International S&T Collaboration on Water Environmental Monitoring and Simulation in the Three Gorges Reservoir Region, College of Resources and Environment and Chongqing Key Laboratory of Tree Germplasm Innovation and Utilization, School of Life Sciences, Southwest University, Chongqing 400715, China; xingshuid@outlook.com (X.D.); nsharifullah@gmail.com (S.S.); shuaiwang@swu.edu.cn (S.W.); xinhua.he@uwa.edu.au (X.H.); 2022020@ynau.edu.cn (S.S.)

[2] State Key Laboratory for Managing Biotic and Chemical Threats to the Quality and Safety of Agro-products, Institute of Environment, Resource, Soil and Fertilizers, Zhejiang Academy of Agricultural Sciences, Hangzhou 310021, China; linhui@zaas.ac.cn (H.L.); majw@zaas.ac.cn (J.M.); wangfeng@zaas.ac.cn (F.W.)

[3] Department of Land, Air and Water Resources, University of California at Davis, Davis, CA 90616, USA; huahe@ucdavis.edu (X.H.)

[4] School of Biological Sciences, University of Western Australia, Perth 6009, Australia; xinhua.he@uwa.edu.au (X.H.)

* Correspondence: xinhua.he@uwa.edu.au (X.H.), Tel.: 86-18723289058 and majw@zaas.ac.cn (J.M.), Tel: 86-13857166772

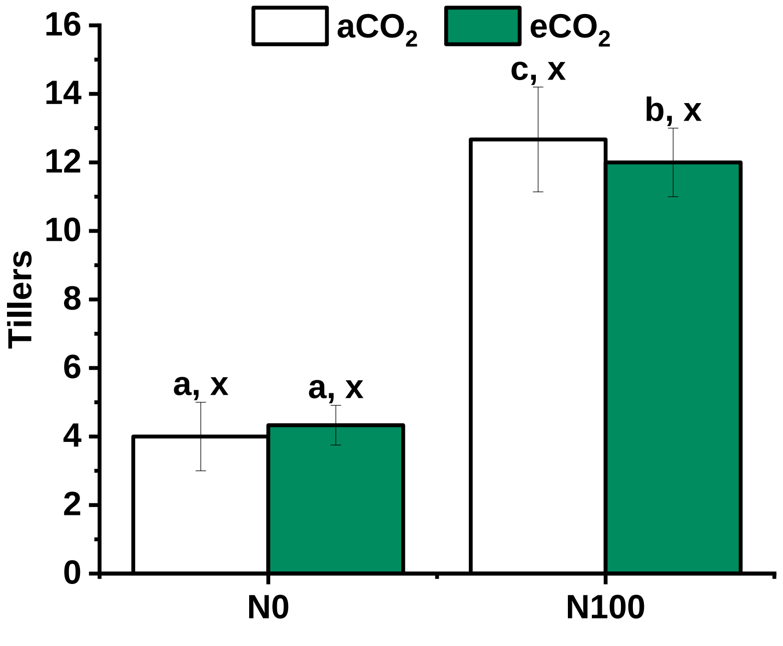

Figure S1. Effects of CO_2 and N supply on tillers of 5-month-old wheat plants at harvest. Abbreviations: aCO_2, atmospheric CO_2; eCO_2, elevated CO_2; N0, no N supply; N100, 100 mg N kg^{-1} DW soil. Data are means ± SE (n = 3). Lower-case letters above the bars indicate significant differences between N supply for the same CO_2 treatment (a, b) and between CO_2 concentrations for the same N treatment (x, y) at $P < 0.05$.

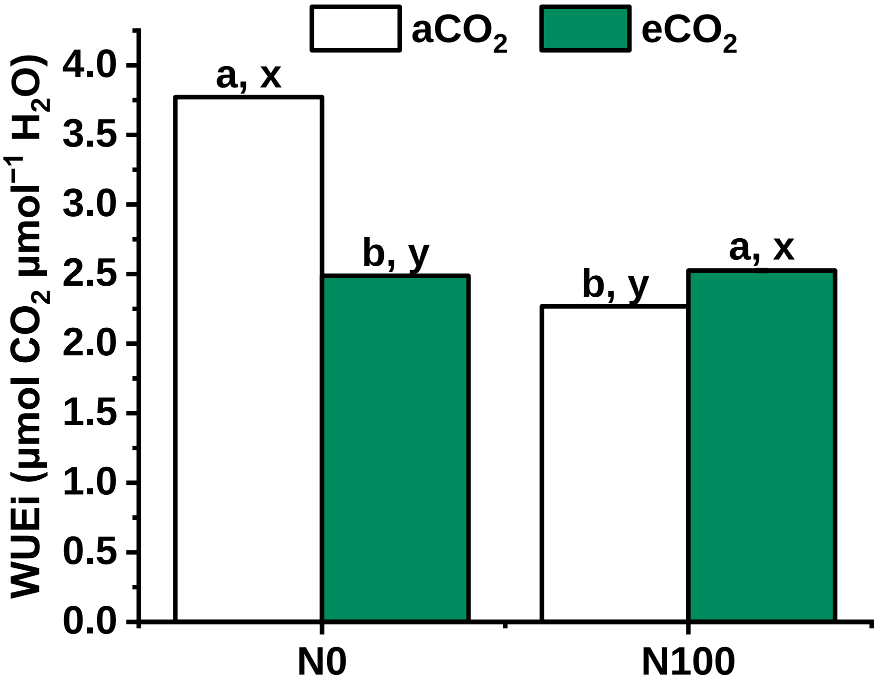

Figure S2. Effects of CO_2 and N supply on WUEi of wheat plants at the booting stage. Abbreviations: aCO_2, atmospheric CO_2; eCO_2, elevated CO_2; N0, no N supply; N100, 100 mg N kg^{-1} DW soil; WUEi, the instantaneous water use efficiency at the leaf scale (μmol CO_2 μmol^{-1} H_2O). Data are means ± SE (n = 3). Lower-case letters above the bars indicate significant differences between N supply for the same CO_2 treatment (a, b) and between CO_2 concentrations for the same N treatment (x, y) at $P < 0.05$.

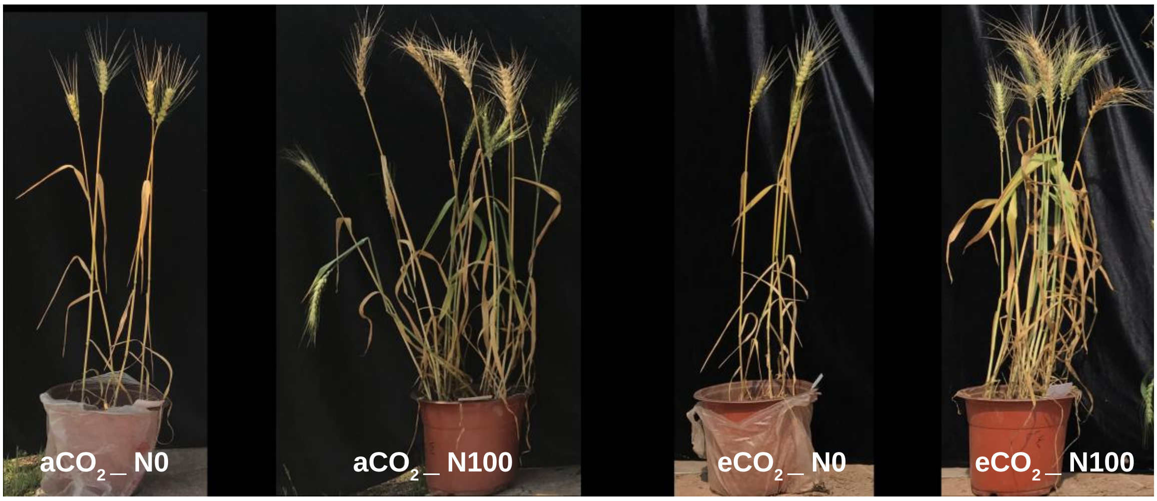

Figure S3. Effects of different CO_2 and N supply on their growth status of 5-month-old wheat plants at harvest. Abbreviations: aCO_2, atmospheric CO_2; eCO_2, elevated CO_2; N0, no N supply; N100, 100 mg N kg^{-1} DW soil.
